# Building a tool to assess malaria surveillance and response capacity in malaria post-elimination contexts: a modified and dual-weighted Delphi approach

**DOI:** 10.1186/s40249-025-01401-w

**Published:** 2025-12-26

**Authors:** Guangyu Lu, Enyu Xu, Yuanyuan Cao, Liying Chai, Zihuan Liao, Jingxia Wang, Taining Sha, Yin Wang, Olaf Müller, Jinkou Zhao, Guoding Zhu, Jun Cao

**Affiliations:** 1 School of Basic Medical Sciences & School of Public Health, Faculty of Medicine, Yangzhou University, Yangzhou, China; 2 Jiangsu Key Laboratory of Zoonosis, Yangzhou University, Yangzhou, China; 3 National Health Commission Key Laboratory of Parasitic Disease Control and Prevention, Jiangsu Provincial Key Laboratory On Parasite and Vector Control Technology, Jiangsu Institute of Parasitic Diseases, Wuxi, China; 4 Center for Global Health, School of Public Health, Nanjing Medical University, Nanjing, China; 5 Yangzhou Center for Disease Control and Prevention, Yangzhou, China; 6 Medical School, Heidelberg Institute of Global Health, Ruprecht-Karls-University, Heidelberg, Germany; 7 The Global Fund to Fight AIDS, Tuberculosis and Malaria, Geneva, Switzerland

**Keywords:** Malaria, Elimination, Globalization, Prevention, Reintroduction, Surveillance, Assessment

## Abstract

**Background:**

Sustaining the elimination of malaria requires robust surveillance to prevent reintroduction, but standardized frameworks for assessing the surveillance capacity of a country post-elimination are lacking. This study aims to develop a standardized framework for assessing malaria surveillance and response capacity in countries that have eliminated malaria.

**Methods:**

We developed a malaria surveillance and response assessment framework through a three-stage process. First, two systematic reviews were conducted to identify indicators used in post-elimination settings worldwide and specifically in China. The candidate indicators were refined through expert panel discussions, which yielded 45 indicators across six domains. Next, a modified two-round Delphi process was conducted, involving 30 experts in epidemiology, disease control, and public health from diverse institutions and administrative levels. The experts rated the importance and feasibility of the indicators using structured questionnaires and then engaged in group discussions to contextualize the findings. Indicator weights were determined using a combined analytic hierarchy process (AHP) and entropy methods.

**Results:**

The systematic reviews and expert consultations identified 45 candidate indicators. After two rounds of expert consultation, a framework comprising 34 indicators across six domains for assessing malaria surveillance and response capacity in post-elimination settings was developed. The weights of the six domains are as follows: surveillance system coverage and performance (0.240); the quality and use of the surveillance data (0.3710); the functioning of the information management system (0.0973); the availability and adequacy of resources (0.0375); early diagnosis and treatment (0.1571); and quality control supervision and training (0.0973). The expert authority coefficient (*Cr*) values of the first and second rounds were 0.777 and 0.895 respectively. Of the 34 indicators, the proportion of confirmed cases with completed epidemiological investigations and submitted reports (0.1153) and the interval between the first medical visit and diagnosis (0.1131) had the highest weights.

**Conclusion:**

This consensus-based framework provides a standardized tool for evaluating malaria surveillance and response capacity in post-elimination settings. Adoption of the framework could help countries monitor and improve their systems to sustain elimination of the disease, mitigate reintroduction risks, and support global malaria eradication efforts.

**Graphical Abstract:**

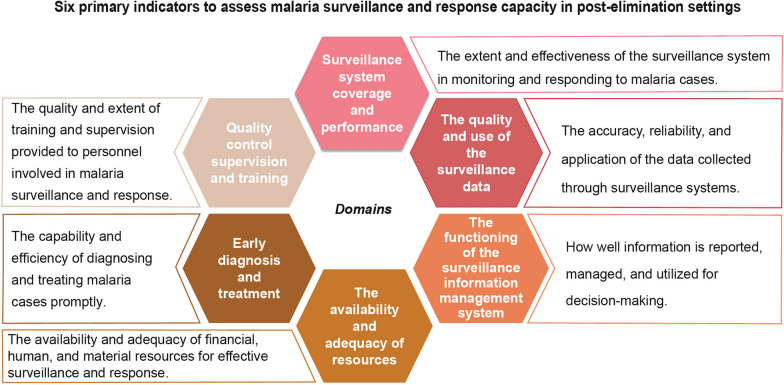

**Supplementary Information:**

The online version contains supplementary material available at 10.1186/s40249-025-01401-w.

## Background

While malaria remains a global health challenge, more than 50 countries have eliminated the disease in the past century [1]. However, increasing international travel has led to an increase in imported malaria cases in malaria-free regions [2–4]. These cases, which are defined by the World Health Organization (WHO) as originating from a malarious area outside the country of diagnosis, pose a significant risk of re-establishing the disease in the latter through local mosquito vectors [5]. The WHO emphasizes the high importance of the implementation of robust surveillance and response systems for the prevention of re-establishment (POR) of malaria transmission [6]. These systems include both passive and active case detection, as well as entomological surveillance, to ensure early detection and timely response to malaria cases and vectors, thereby maintaining the country’s malaria-free status [6, 7].

The WHO emphasizes routine assessments of these surveillance systems to ensure that they meet POR objectives [7, 8]. Although various assessment tools exist [9–11], most focus on elimination-phase settings rather than post-elimination contexts [12]. This gap leaves malaria-free countries without standardized metrics to evaluate their surveillance capacity, potentially jeopardizing their hard-won elimination statuses.

China's recent malaria-free certification (June 2021) [13] and its innovative “1-3-7” surveillance strategy (i.e., requiring case reporting within 1 day, investigation within 3 days, and response within 7 days) have provided valuable experience for developing post-elimination assessment tools [14, 15]. As China's surveillance focus shifted from case reduction to zero-transmission maintenance, the need for appropriate surveillance evaluation metrics has become apparent.

The present study emerged from China's urgent need to assess its post-elimination surveillance capacity. Recognizing the lack of suitable existing tools, we developed and validated a comprehensive assessment framework specifically designed for malaria-free contexts. Our tool addresses critical gaps in current approaches by focusing on post-elimination surveillance challenges and incorporating lessons from China's successful elimination program, providing standardized metrics for malaria surveillance comparable across post-elimination regions and emphasizing practical implementation for ongoing POR efforts.

The resulting framework serves as an evidence-based tool that malaria-free countries can use to monitor and strengthen their surveillance systems, helping sustain elimination achievements amid growing importation risks. By sharing China's experience and this new assessment approach, we aim to support global malaria elimination goals while addressing a critical gap in the evaluation of post-certification surveillance.

## Methods

**Fig. 1:**
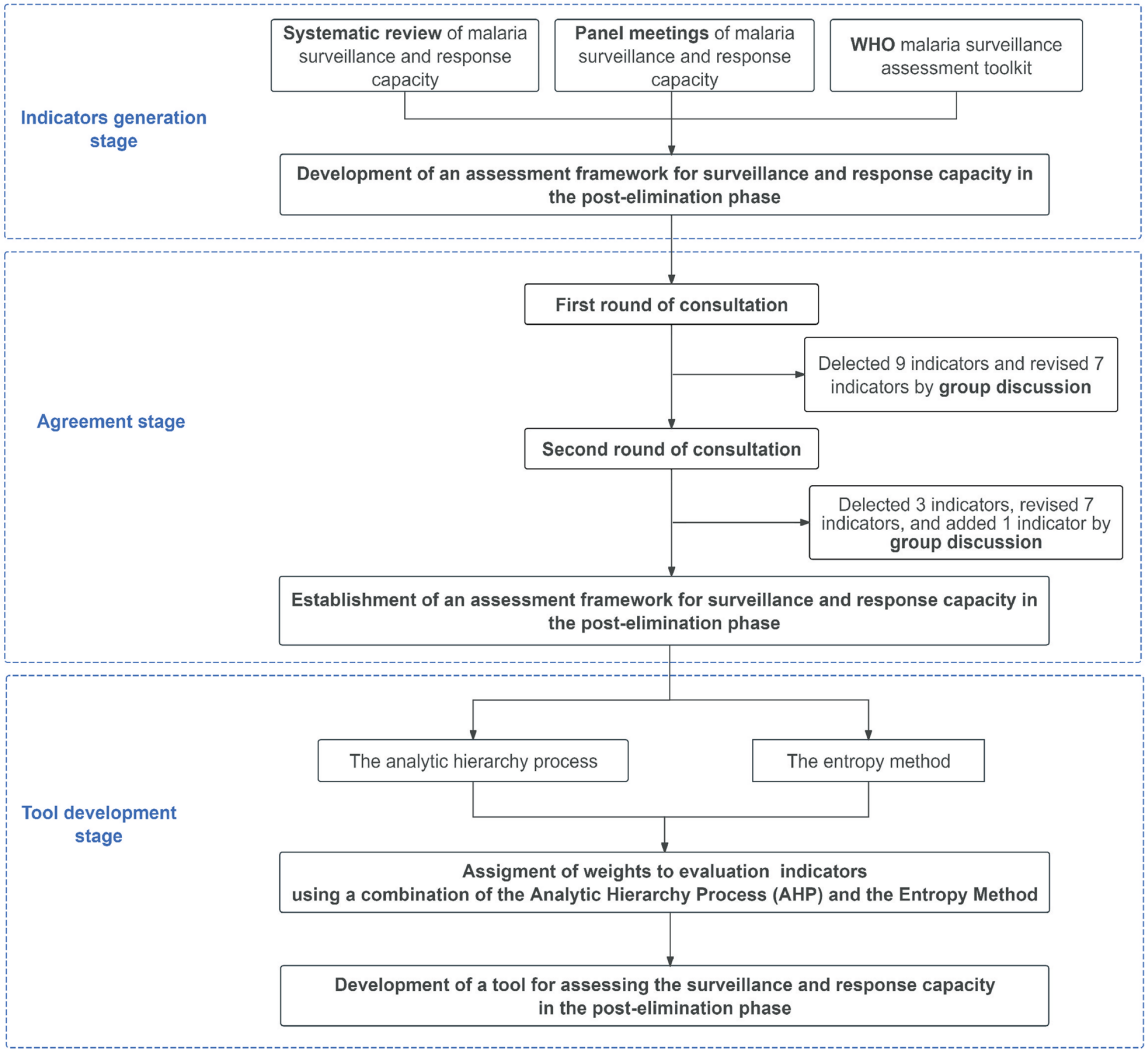
Flowchart of the development of the assessment tool for malaria surveillance and response capacity in post-elimination settings

This study was conducted in accordance with the Guidance on Conducting and REporting DElphi Studies guidelines (Supplemental Appendix 1) [16]. The development of the evaluation tool comprised three stages, as shown in the flowchart in Fig. 1: (1) indicator generation stage: identification of indicators through systematic reviews and expert panel meetings; (2) agreement stage: consensus-building through Delphi surveys to determine the importance of the indicators; and (3) tool development stage: weight determination and tool finalization.

### Indicator generation stage

We first conducted two comprehensive systematic reviews to collect all potential indicators used for assessing malaria surveillance in pre- and post-elimination settings globally [17–19]. The search strategy, review procedures, and full list of extracted indicators are summarized in Supplemental Appendix 2. Indicators from existing official documents were integrated through the panel meeting for developing a proposed framework to assess surveillance and response capacity in the post-elimination phase.

### Agreement stage

During the Delphi study, we adopted a mixed-methods approach with both quantitative and qualitative data. Each round consisted of a quantitative survey in which experts rated indicators using Likert scales, followed by a qualitative group discussion. These discussions were conducted after each survey to contextualize the data collected from the surveys.

#### Study coordination and team composition

The study was coordinated by the WHO Collaborating Centre for Research and Training on Malaria Elimination at the Jiangsu Institute of Parasitic Diseases. The study team, comprising epidemiologists, entomologists, policy makers, malaria researchers, and global health researchers, and its broad network helped the team recruit a diverse and authoritative panel.

#### Expert selection

The experts included those engaged in malaria surveillance and response, whether in the field, in policy-making, or in research contexts. All participants had at least five years of experience in at least one of the following areas of specialization: (1) malaria surveillance program design and development; (2) malaria surveillance implementation; (3) malaria surveillance research; and (4) management of imported malaria cases at customs. Together, these experts provided comprehensive opinions and actively engaged in both rounds of consultations. In contrast to previous Delphi consultations, study participants were recruited from different administrative levels of the health system in China to assess the importance and feasibility of the tool. The detailed process used to contact and select the experts is presented in Supplemental Appendix 3.

#### Study duration and Delphi consultation

Data collection was conducted from June to December 2023. On the basis of a preliminary assessment framework, we developed a Delphi questionnaire comprising (1) a cover letter describing the study background, objectives, and key evidence from the systematic reviews and the WHO malaria surveillance toolkit [20]; (2) expert demographic information; (3) questionnaire instructions; and (4) Delphi consultation content. The experts were briefed via telephone before the first round of Delphi. In each round, they rated each indicator’s importance and feasibility (1 = very unimportant/unfeasible, 5 = very important/feasible) and their familiarity with malaria surveillance (1 = very unfamiliar, 5 = very familiar) while also providing open-ended feedback (Supplemental Appendix 4). In two rounds, the results were aggregated and shared, and the experts re-evaluated the indicators until a consensus was achieved (mean importance ≥ 4/5 with stable agreement) [21]. Indicators were screened using the boundary value method: those scoring above the mean-standard deviation (SD) for the top-score frequency and arithmetic mean, and below the mean + SD for the coefficient of variation were retained; the other indicators were reviewed in focus group discussions [22]. Detailed thresholds and statistical methods are provided in Supplemental Appendix 3.

#### Feedback between rounds

After each Delphi round, a structured feedback report was shared with all the experts [23]. This report consolidated three key elements of the Delphi consultation: the expert’s own prior ratings, a statistical summary of the group’s responses (including measures of central tendency and dispersion), and anonymized qualitative comments provided by fellow experts [24]. The feedback was provided to guide iterative refinement of the assessment tool. Indicators that consistently showed low consensus ratings were reconsidered during a final expert consultation, after which they were revised or removed. During this process, insights from the written comments helped clarify indicator definitions and practical applicability.

### Tool development stage

We employed a combination of subjective and objective methods to establish the indicator weights. Subjective weights were derived using the analytic hierarchy process (AHP), in which the indicators were organized into a hierarchical structure and evaluated through pairwise comparisons based on the Saaty criteria (Supplemental Appendix 3 Tables S2, S3) [25]. Objective weights were calculated using the entropy method, which quantifies the information content of each indicator on the basis of the variability in actual field data collected from 13 cities (Supplemental Appendix 3 Table S4) [26]; indicators with greater variability were assigned higher entropy weights [26]. The final composite weight for each indicator was obtained by multiplying its AHP weight and entropy weight; if the entropy weight was zero, the AHP weight was retained as the composite weight. Finally, all the composite weights were normalized to sum to 1 [27]. By multiplying the two weights, the theoretical importance as judged by the experts is balanced with the empirical discriminative power observed in the field, increasing the robustness and practical relevance of the weighting scheme.

### Ethis approval and consent to participate

The study protocol was reviewed and approved by the Yangzhou University Ethics Committee (Ethics Number: YZUHL20220077). All participants consented to the study with the knowledge that anonymity would not be preserved (neither during the focus group session nor during the Delphi study, to enable follow-up). A final, open virtual dissemination meeting was held in March 2024 to share the results and receive feedback on considerations for implementation from a wider audience. No patients or members of the public were involved in the design, conduct, reporting, or dissemination of this research.

## Results

### Indicator generation stage

#### Systematic review

Two systematic reviews were conducted to identify indicators for assessing the risk of malaria reintroduction, which were subsequently categorized into six domains: environmental/meteorological, historical epidemiology, vector-related, sociodemographic, surveillance/response, and population migration factors. Thirty-eight surveillance/response indicators were extracted (Supplemental Appendix 2 Table S3).

#### Panel meeting

**Fig. 2:**
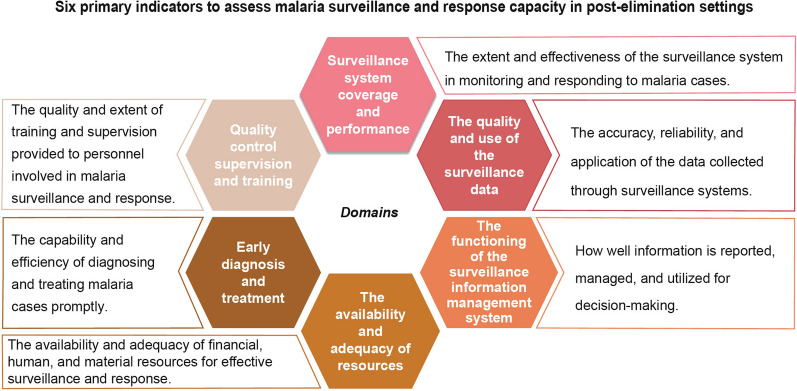
Six domains to assess malaria surveillance and response capacity in post-elimination settings

The study team reviewed indicators from the systematic reviews and the WHO toolkit [20], ultimately arriving at 45 surveillance/response indicators across six domains. (Fig. 2). (Supplemental Appendix 3 Table S5).

### Agreement stage

Thirty out of 40 invited participants (75%) contributed to the consensus process. The 30 experts were from different administrative levels and had diverse specializations in fields including epidemiology, infectious disease prevention and control, imported infectious disease quarantine, and vector biology and control. This sample provided a highly diverse set of perspectives from the relevant participant groups. The expert panel was primarily composed of individuals with master’s degrees and doctor of philosophy (PhD) degrees (15/30, 50%). Sixty percent (18/30) of the experts held senior professional titles. The members of the panel had been practising as specialists for a median of 22 years (interquartile range: 15–27.8). The characteristics of the expert panel are detailed in Table 1.

**Table 1:** Delphi expert characteristics in developing the malaria surveillance and response capacity assessment framework

Characteristic	Number	Percentage (%)
Gender		
Male	19	63.3
Female	11	36.7
Age (years)		
30–39	5	16.7
40–49	18	60.0
50–60	7	23.3
Level of education		
Undergraduate	15	50.0
Master degree	8	26.7
PhD degree	7	23.3
Professional title		
Junior title	1	3.3
Intermediate title	4	13.3
Associate senior title	7	23.3
Senior title	18	60.0
Expertise/working background		
Academia	5	16.7
Entomologist	2	6.7
Customs	3	10.0
Provincial-level CDC^a^	2	6.7
Municipal/city-level CDC	15	50.0
County-level CDC	1	3.3
Policy maker	2	6.7
Years of working experience		
< 10	3	10.0
10–19	8	26.7
20–29	13	43.3
30–40	6	20.0

^a^CDC: Centers for Disease Control and Prevention

#### Results of the Delphi study

Two rounds of Delphi assessments (June–December 2023) yielded 100% response rates, as shown in Table 2. The authority, coordination, and concentration among the expert opinions in the two rounds of Delphi questionnaires for the development of a malaria surveillance and response capacity assessment framework in post-elimination settings are shown in Tables 2 and 3.

**Table 2:** Degree of positivity and authority among expert opinions in the first and second rounds of the Delphi assessment for developing the malaria surveillance and response capacity assessment framework in post-elimination settings

Round	Response rate (%)	Cs^a^	Ca^b^	Cr^c^
The first round of Delphi	100	0.846	0.708	0.777
The second round of Delphi	100	0.868	0.922	0.895

^a^Cs: familiarity with the indicators

^b^Ca: judgment basis for judging the indicators

^c^Cr: authority coefficient

**Table 3:** Degree of coordination and concentration among expert opinions in the first and second rounds of the Delphi assessment for developing the malaria surveillance and response capacity assessment framework in post-elimination settings

	The first round of Delphi		The second round of Delphi	
	Importance	Feasibility	Importance	Feasibility
*CV*^*a*^	0.127	0.156	0.103	0.145
Kendall’s *W*^b^	0.205	0.149	0.263	0.242
*χ*^c^	307.099	174.377	328.151	363.317
*P*	< 0.001	< 0.001	< 0.001	< 0.001

^a^*CV:* the coefficient of variation

^b^*W*: Kendall’s *W* coefficient. A higher value indicates greater consensus among experts regarding the indicator and thus a higher degree of agreement

^c^*χ*^*2*^: Statistic of the chi-square test for assessing the significance of Kendall’s *W* coefficient. A higher *χ*^*2*^ value indicates stronger evidence against the null hypothesis (which typically assumes no agreement among experts)

**Fig. 3:**
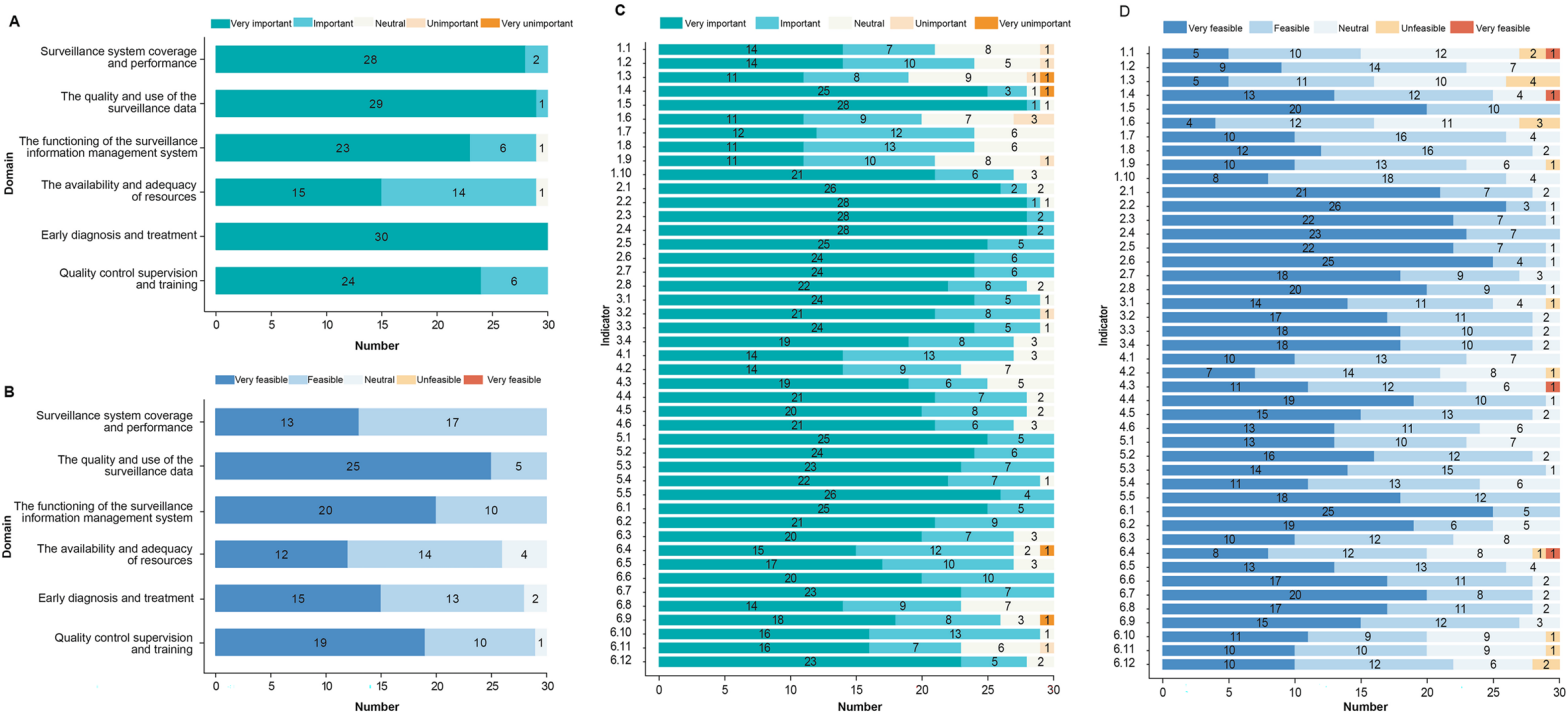
Experts’ opinions about the importance and feasibility of each domain and indicator in the first round of the Delphi assessment. **A** Importance of each domain; (**B**) feasibility of each domain; (**C**) importance of each indicator; (**D**) feasibility of each indicator

Among the outcomes presented in the first round of the Delphi assessment, 25/45 indicators were rated as “very important” by more than 70% of the participants. All indicators belonging to the domains “the quality and use of the surveillance data” and “early diagnosis and treatment” were rated as “very important” by ≥ 70% of the participants. Only 7/45 indicators were rated as “very feasible” by ≥ 70% of the participants, 6 of which were in the domain “the quality and use of the surveillance data” (Fig. 3). Nine indicators were deleted, and 7 indicators were revised after screening according to the boundary values and group discussions (Supplemental Appendix 3 Table S6). Overall, 36 prevention indicators were presented for rating in the second round of the Delphi assessment.

**Fig. 4:**
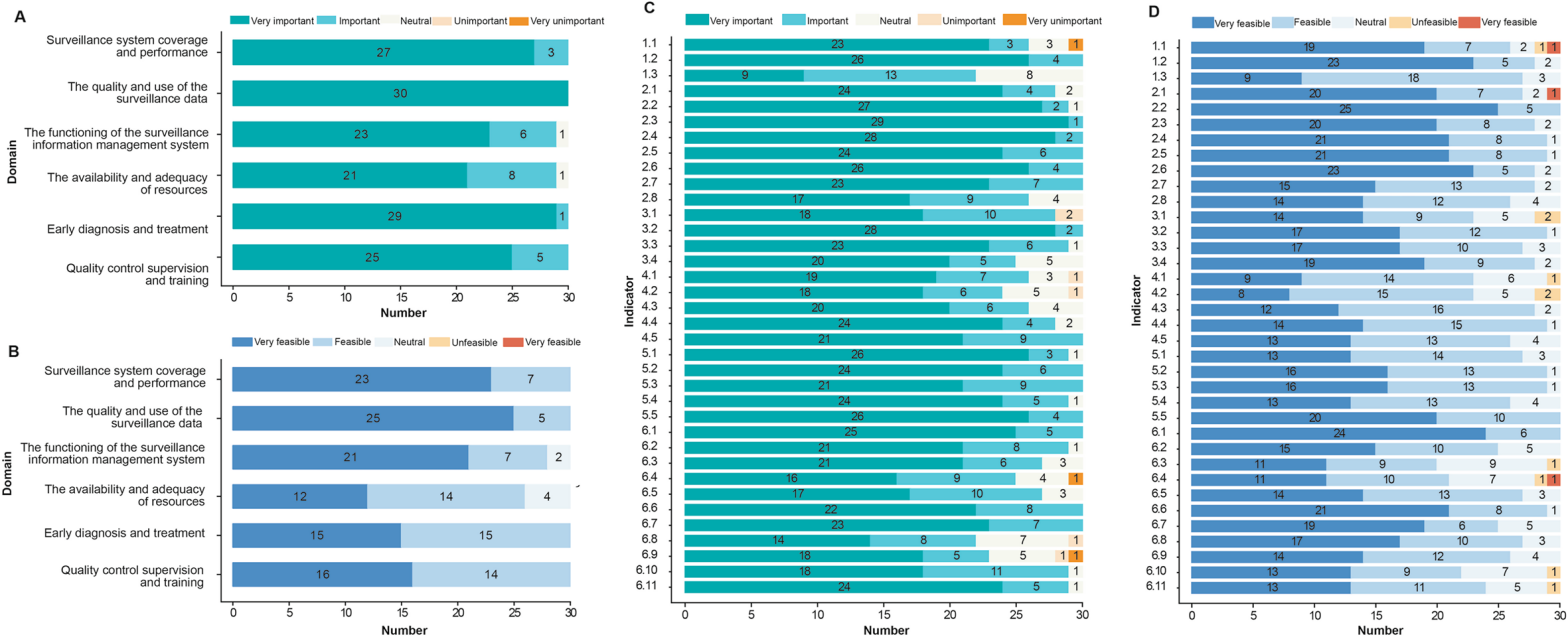
Experts’ opinions about the importance and feasibility of each domain and indicator in the second round of the Delphi assessment. **A** Importance of each domain; (**B**) feasibility of each domain; (**C**) importance of each indicator; (**D**) feasibility of each indicator

During the second round of the Delphi assessment, 24/36 indicators were rated as “very important” by ≥ 70% of the participants (Fig. 4); moreover, 26/36 indicators were rated as “very important” or “important” by ≥ 90% of the participants. The indicators “regularity of vector surveillance activities”, “number of malaria awareness-raising materials distributed”, and “frequency of public lectures on malaria” were rated as having the lowest importance. Seven of the 36 indicators were rated as “very feasible” by ≥ 70% of the participants. A total of 4/8 indicators of “the quality and use of the surveillance data” domain were rated as “very feasible” by  ≥ 70% of the participants (Fig. 4). Three indicators were deleted, 7 indicators were revised, and 1 indicator was added on the basis of the boundary values and group discussion (Supplemental Appendix 3 Table S6).

Ultimately, as shown in Figure S1 in the Online Supplemental Appendix 3, 34 indicators in six domains were selected for developing the assessment framework.

### Tool development stage

The weights of both the domains and indicators are presented in Table 4 and Supplemental Appendix 3 Table S7. A user-friendly Excel-based tool was developed to easily calculate scores representing surveillance and response capacity (Supplemental Appendix 5).

**Table 4:** Domain weights for the assessment of malaria surveillance and response in post-elimination settings

The domain	Weights
Surveillance system coverage and performance	0.2400
The quality and use of the surveillance data	0.3710
The functioning of the surveillance information management system	0.0973
The availability and adequacy of resources	0.0375
Early diagnosis and treatment	0.1571
Quality control supervision and training	0.0973

## Discussion

Despite the importance of malaria surveillance assessment in post-elimination settings, existing assessment tools remain inadequate. Our study provides a new indicator-based framework that emphasizes adaptability to shifting epidemiology and local contexts. By incorporating the perspectives of stakeholders from multiple disciplines, we developed a practical tool to help policymakers evaluate and strengthen surveillance systems, mitigating the risk of malaria re-establishment.

Timely detection of imported malaria cases is critical in post-elimination settings, making the interval between the first medical visit and diagnosis a key surveillance indicator. Delays in care seeking persist even after symptom onset (e.g., fever), with factors contributing to this delay showing regional variability [28–31]. In China, most imported cases occur among labourers returning from malaria-endemic regions in Africa and Southeast Asia, where low awareness of malaria symptoms among both patients and healthcare providers often delays diagnosis [32]. In contrast, Western countries primarily see imported cases among travellers visiting friends/relatives (VFRs), immigrants, and refugees. Among these groups, delays stem from a low perceived risk of malaria, limited healthcare access (particularly for uninsured or nonnative populations), and frequent misdiagnosis as influenza or other common illnesses [33–36]. These systemic delays increase the risk of severe outcomes and, potentially local transmission. The time-to-diagnosis metric thus serves as an objective, actionable measure of surveillance performance, highlighting gaps in case detection and provider readiness. Its inclusion in post-elimination assessment tools reflects the need for targeted interventions addressing context-specific barriers to prompt diagnosis.

While the experts recognized the critical need to assess knowledge on malaria prevention among high-risk groups, they rated the feasibility of data collection as low. For example, in China, migrant workers face particular vulnerability because of limited health literacy, poor understanding of the risk of transmission, and inconsistent use of protective measures [28, 37]. These gaps often lead to dangerous behaviours, such as delayed care seeking, incomplete communication of travel history, and self-medication, increasing the risks of severe outcomes and local transmission upon return [37–39]. In response, China implemented a multisectoral prevention program involving travel health centres, labour agencies, and community organizations [40]. However, significant challenges in reaching highly mobile populations and evaluating the effectiveness of this intervention remain, while the lack of standardized assessment tools further complicates accessibility to monitoring and prevention knowledge across diverse groups, such as migrant workers and travellers. Future efforts must prioritize (1) developing validated measurement instruments, (2) improving health education accessibility for mobile populations, and (3) increasing participation in prevention programs. These steps are essential for mitigating importation risks and preventing secondary transmission in elimination settings.

Despite its importance for malaria control, mosquito surveillance scored low in terms of feasibility and significance in our tool. Existing programs often lack sufficient resources, long-term planning, and technical capacity—particularly in elimination settings—resulting in unreliable data that poorly inform public health decisions [41, 42]. These factors may lead to a disconnect between entomological and epidemiological indices, wherein entomological data inadequately inform disease control efforts [43]. To address these gaps, we recommend establishing sentinel surveillance sites to generate high-quality, targeted data. Additionally, countries should develop cost-effective, strategic plans to maintain essential vector monitoring post-elimination [43]. Such approaches would improve the utility of mosquito surveillance while optimally leveraging limited resources, ensuring that entomological data remain actionable for sustaining malaria-free status.

Data quality emerged as the highest-weighted domain, reflecting its critical role in effective surveillance. Complete, valid case reporting remains essential for accurate monitoring, particularly in post-elimination settings where data validity concerns persist [44, 45]. Requiring timely reporting through legal frameworks and thorough case investigations would strengthen the reliability of the surveillance; however, contextual challenges across all health system levels—from national policies to local reporting—must be considered before such reporting can be implemented [46]. Given potential challenges in collecting the relevant data, particularly in resource-limited settings, users could consider prioritizing high-weight indicators or drawing on alternative or proxy data sources. Robust data quality mechanisms are fundamental for maintaining the elimination status of the disease and guiding targeted interventions when new cases emerge.

The WHO’s 2022 malaria surveillance toolkit includes 79 indicators, of which 49 are priorities for eliminating countries and 40 are for burden-reduction settings; our post-elimination-specific tool expands on this toolkit by introducing new, critical indicators addressing two key challenges: (1) early detection/treatment of imported cases and (2) knowledge of malaria among high-risk groups. These additions reflect the distinct epidemiological needs of malaria-free countries, where POR requires focused surveillance of importation risks and vulnerable populations [47]. As the dynamics of malaria evolve post-elimination, tailored assessment tools become essential for maintaining elimination status and guiding targeted interventions.

### Global health implications

This study introduces a dual-weighted tool specifically designed to assess malaria surveillance and response capacities in post-elimination contexts to address relevant gaps, such as the generic nature of existing tools that are often unfit for this purpose. Countries that have eliminated malaria—such as Chile, China, Sri Lanka, and Cabo Verde—have adopted a range of strategies to maintain vigilance in the postelimination phase that typically include rapid investigations and responses to imported cases, strong cross-sectoral coordination, targeted risk mapping, and greater monitoring at points of entry [17, 48–50]. To this end, our dual-weighted framework identifies system weaknesses and aims to harmonize best practices, reducing re-establishment risks and facilitating global malaria-free sustainability.

To further validate and refine the assessment tool, multicountry pilot studies should be planned. For example, three to five countries that represent diverse health systems, malaria transmission histories, and geographical regions (e.g., Southeast Asia, Europe, and the Americas) could be selected. A mixed-methods design could be employed. Quantitatively, the tool would be administered to calculate surveillance capacity scores and tested for internal consistency and reliability, while qualitatively, focus group discussions and in-depth interviews with national malaria program managers and frontline staff will be conducted to assess the tool's usability, relevance, and feasibility. To further improve the universality of the tool, participating countries may consider convening regional expert panels to recalibrate AHP weights or adjust indicator definitions so that the scoring framework aligns with local priorities and system structures. This adaptable validation approach would support broader uptake and ensure that the tool functions effectively across heterogeneous post-elimination environments.

### Strengths and limitations

The strengths of this study include the use of a rigorous Delphi consensus method and the incorporation of experts and frontline implementers with diverse specialties and administrative levels. Our tool uniquely combines subjective (AHP) and objective (entropy) weighting of indicator importance and feasibility. However, certain limitations should also be acknowledge. First, potential homophily bias persists despite the systematic selection of the participants. Second, the expert panel was drawn from a single country, which may affect the generalizability of the subjective weightings to other settings. Although the tool has been validated in the Chinese context, its applicability to other epidemiological and health system contexts requires further verification. In addition, the collection of some indicators may face feasibility challenges in resource-limited settings, particularly in countries in the post-elimination phase. Addressing such challenges will require strong government commitment and multisector collaboration. For example, the awareness and knowledge of malaria prevention among migrant labourers in malaria-endemic countries could be followed by collaboration with labour-export companies before their departure. Furthermore, operational research is needed to evaluate the tool’s performance and adaptability across different elimination settings.

## Conclusion

We established a consensus-based assessment tool for malaria surveillance and response capacity in areas where malaria has been eliminated with the aim of POR of the disease. The tool is supported by experts from multiple specialties and real-world implementers from different administrative levels. Adoption of this framework can support countries in monitoring, improving, and sustaining malaria elimination and inform global efforts to prevent malaria re-establishment.

## Supplementary Information

Supplementary Material 1.

Supplementary Material 2.

Supplementary Material 3.

Supplementary Material 4.

Supplementary Material 5.

## Data Availability

All data relevant to the study are included in the article or uploaded as online supplemental information.

## Abbreviations

AHP: Analytic Hierarchy Process
CDC: Centers for Disease Control and Prevention
Ca: The judgement basis of the indicators
*C**r*: The authoritative coefficient
Cs: The familiarity with the indicators
*CV*: The coefficient of variation
ECDC: European Centre for Disease Prevention and Control
POR: Prevention of re-establishment
PhD: Doctor of Philosophy
SD: Standard deviation
VFRs: Visiting friends/relatives
WHO: World Health Organization
