# Supplementary material for: Building a tool to assess malaria surveillance and response capacity in malaria post-elimination contexts: a modified and dual-weighted Delphi approach
Source: Infect Dis Poverty. 2025 Dec 26;14:127. doi: 10.1186/s40249-025-01401-w (PMC12742199; doi:10.1186/s40249-025-01401-w)
Supplement: Supplementary file 1 — Supplementary Material 1. [file 40249_2025_1401_MOESM1_ESM.pdf]

Appendix 1: The Guidance on Conducting and REportingDElphiStudies (CREDES)

Recommendations for the Conducting and REporting of DElphi Studies (CREDES)[1].

"YES" infers the quality criterion has been met in the current study.

Rationale for the choice of the Delphi technique

1. Justification. The choice of the Delphi technique as a method of systematically collating expert consultation and building consensus needs to be well justified. When selecting the method to answer a particular research question, it is important to keep in mind its constructivist nature. YES,P2-P4

Planning and design

2. Planning and process. The Delphi technique is a flexible method and can be adjusted to the respective research aims and purposes. Any modifications should be justified by a rationale and be applied systematically and rigorously. YES,P2-P4
3. Definition of consensus. Unless not reasonable due to the explorative nature of the study, an a priori criterion for consensus should be defined. This includes a clear and transparent guide for action on (a) how to proceed with certain items or topics in the next survey round, (b) the required threshold to terminate the Delphi process and (c) procedures to be followed when consensus is (not) reached after one or more iterations. YES,P4

Study conduct

4. Informational input. All material provided to the expert panel at the outset of the project and throughout the Delphi process should be carefully reviewed and piloted in advance in order to examine the effect on experts' judgements and to prevent bias. YES, P2-4, supplemental appendix4
5. Prevention of bias. Researchers need to take measures to avoid directly or indirectly influencing the experts' judgements. If one or more members of the research team have a conflict of interest, entrusting an independent researcher with the main coordination of the Delphi study is advisable *Consolidated criteria for reporting qualitative research **Consolidated Standards of Reporting Trials 702 Palliative Medicine 31(8). YES, P3-P4
6. Interpretation and processing of results. Consensus does not necessarily imply the 'correct' answer or judgement; (non)consensus and stable disagreement provide informative insights and highlight differences in perspectives concerning the topic in question. YES, P2-P4
7. External validation. It is recommended to have the final draft of the resulting guidance on best practice in palliative care reviewed and approved by an external board or authority before publication and dissemination. YES, P4

Reporting

8. Purpose and rationale. The purpose of the study should be clearly defined and demonstrate the appropriateness of the use of the Delphi technique as a method to achieve the research aim. A rationale for the choice of the Delphi technique as the most suitable method needs to be provided. YES, P2
9. Expert panel. Criteria for the selection of experts and transparent information on recruitment of the expert panel, sociodemographic details including information on

expertise regarding the topic in question, (non)response and response rates over the ongoing iterations should be reported. YES, P3-P4

10. Description of the methods. The methods employed need to be comprehensible; this includes information on preparatory steps (How was available evidence on the topic in question synthesised?), piloting of material and survey instruments, design of the survey instrument(s), the number and design of survey rounds, methods of data analysis, processing and synthesis of experts' responses to inform the subsequent survey round and methodological decisions taken by the research team throughout the process. YES, P2-P4
11. Procedure. Flow chart to illustrate the stages of the Delphi process, including a preparatory phase, the actual 'Delphi rounds', interim steps of data processing and analysis, and concluding steps. YES, P3
12. Definition and attainment of consensus. It needs to be comprehensible to the reader how consensus was achieved throughout the process, including strategies to deal with non-consensus. YES, P4
13. Results. Reporting of results for each round separately is highly advisable in order to make the evolving of consensus over the rounds transparent. This includes figures showing the average group response, changes between rounds, as well as any modifications of the survey instrument such as deletion, addition or modification of survey items based on previous rounds. YES, P5-P7, supplemental appendix 3
14. Discussion of limitations. Reporting should include a critical reflection of potential limitations and their impact of the resulting guidance. YES, P8-9
15. Adequacy of conclusions. The conclusions should adequately reflect the outcomes of the Delphi study with a view to the scope and applicability of the resulting practice guidance. YES, P8-P9
16. Publication and dissemination. The resulting guidance on good practice in palliative care should be clearly identifiable from the publication, including recommendations for transfer into practice and implementation. If the publication does not allow for a detailed presentation of either the resulting practice guidance or the methodological features of the applied Delphi technique, or both, reference to a more detailed presentation elsewhere should be made (e.g. availability of the full guideline from the authors or online; publication of a separate paper reporting on methodological details and particularities of the process (e.g. persistent disagreement and controversy on certain issues)). A dissemination plan should include endorsement of the guidance by professional associations and health care authorities to facilitate implementation. NOT APPLICABLE.

Reference

1. Junger S, Payne SA, Brine J, Radbruch L, Brearley SG. Guidance on Conducting and REporting DElphi Studies (CREDES) in palliative care: Recommendations based on a methodological systematic review. Palliative Medicine 2017;31:684-706.
