# Supplementary material for: Building a tool to assess malaria surveillance and response capacity in malaria post-elimination contexts: a modified and dual-weighted Delphi approach
Source: Infect Dis Poverty. 2025 Dec 26;14:127. doi: 10.1186/s40249-025-01401-w (PMC12742199; doi:10.1186/s40249-025-01401-w)
Supplement: Supplementary file 2 — Supplementary Material 2. [file 40249_2025_1401_MOESM2_ESM.docx]

**Appendix 2**

**The method and results of systematic reviews**

In the first review, studies that developed or validated a malaria risk prediction model in countries certified as malaria-free were included [1]. The search strategies were developed (online supplemental appendix 2 table S1). We searched four electronic databases, including in PubMed, Web of Science, Cochrane Library, and China National Knowledge Infrastructure (CNKI), along with reference lists of publications, from 2008 to March, 2023 (online supplemental appendix 2 figure S1). In the systematic review that assessed the risk of malaria reintroduction in countries certified as malaria-free, our searches yielded 10065 records, with 8772 remaining after duplicate publications were removed. Of these, 8424 records were excluded after title and abstract screening, and the remaining 348 papers were obtained. Full-text screening resulted in the exclusion of an additional 338 records. Therefore, 10 articles that reported on 11 malaria reintroduction risk prediction models in areas where malaria has been eliminated were included (Online Supplemental Appendix 2 figure S1).

**Table S1.** The search database and strategy for the risk of malaria re-introduction in countries certified malaria-free.

| **Database** | **Search terms** |
| --- | --- |
| Web of science | #1 TS=(Malaria OR Malaria, Vivax OR Malaria, Falciparum OR acute malaria)  #2 TS=(Prediction OR Predictive model OR Prediction model OR Risk prediction OR Risk score OR Risk calculation OR Risk assessment)  #3 #1AND #2 |
| PubMed | #1 (((("Malaria"[Mesh]) OR ("Malaria, Vivax"[Mesh])) OR ("Malaria, Falciparum"[Mesh])) OR ("Acute malaria" [Supplementary Concept])) OR (malaria[Title/Abstract])  #2 ((((((Prediction[Title/Abstract]) OR (Prediction model[Title/Abstract])) OR (Predictive model[Title/Abstract])) OR (Risk prediction[Title/Abstract])) OR (Risk score[Title/Abstract])) OR (Risk calculation[Title/Abstract])) OR (Risk assessment)  #3 #1 AND #2 |
| Cochrane | #1 (Malaria OR Malaria, Vivax OR Malaria, Falciparum OR Acute malaria OR malaria):kw  #2 (Prediction OR Predictive model OR Prediction model OR Risk prediction OR Risk score OR Risk calculation OR Risk assessment):kw  #3 #1 AND #2 |
| China National Knowledge Infrastructure Database (CNKI) | #1 Topics: malaria + Malaria, Vivax + Malaria, Falciparum  #2 Topics: 'forecasting*' + risk forecasting + risk scoring + risk calculation+ risk assessment + forecasting models  #3 #1 AND #2 |


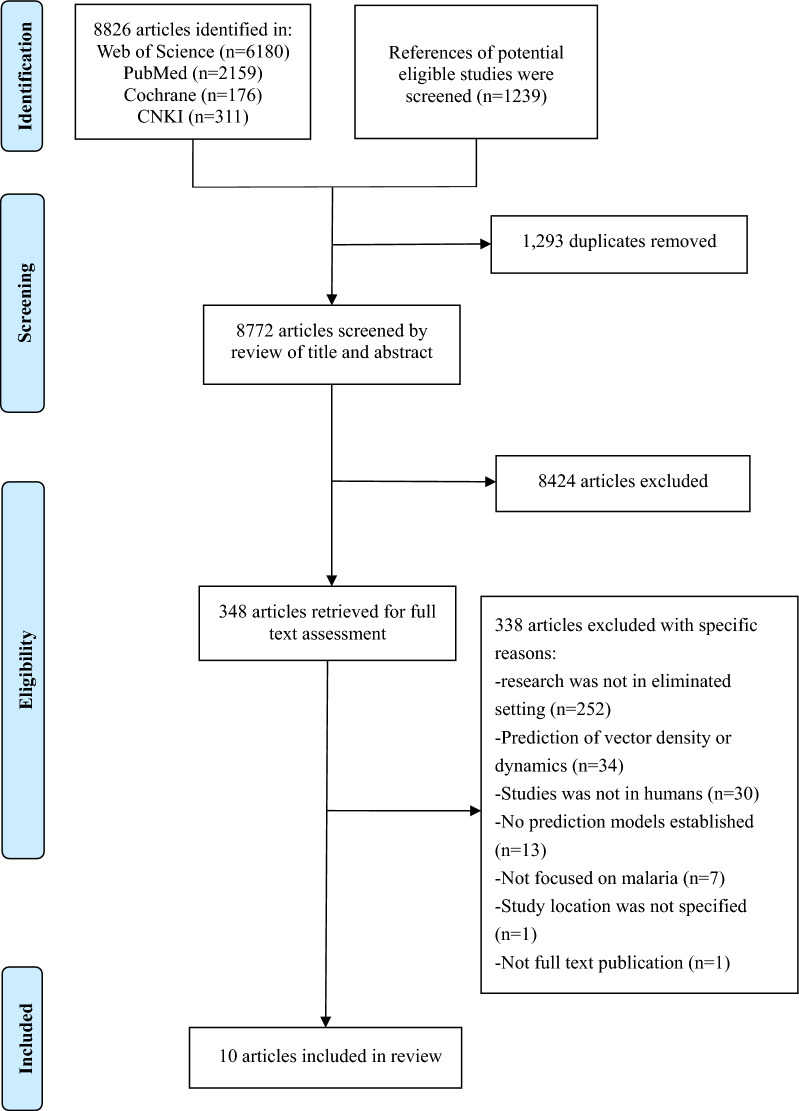


**Figure S1.** A summary flow of study selection for research on malaria re-introduction risk in malaria-free certified countries.

In the second review, studies that assessed the local transmission of malaria or the risk of reintroduction in China since 2010 were included [2]. These search strategies were developed (online supplemental 2 table S2). We searched 6 electronic databases, including in English databases (such as PubMed, Web of Science, Cochrane Library), and in Chinese databases including China National Knowledge Infrastructure (CNKI), China Science and Technology Journal Database (VIP), and Wanfang database, from 2010 to April, 2023 (online supplemental appendix 2 figure S2). In the systematic review that assessed the risk of local malaria transmission and reintroduction in China from pre-elimination to elimination, our searches yielded 8124 records, with 6339 remaining after duplicate publications were removed. Following title and abstract screening, 6090 records were excluded, and the remaining 251 papers were obtained. Full-text screening resulted in the exclusion of an additional 198 records. The remaining 53 articles that reported on 55 malaria risk prediction or assessment models in China from 2010 to 2023 were included (Online Supplemental Appendix 2 Figure S2).

**Table S2.** The search database and strategy for the risk of malaria local transmission and re-introduction in China from the pre-elimination to elimination phases.

| **Database** | **Search terms** |
| --- | --- |
| PubMed | #1 (("Malaria"[Mesh]) OR ("Malaria, Vivax"[Mesh])) OR ("Malaria, Falciparum"[Mesh])) OR (malaria[Title/Abstract]))  #2 (("Disease Transmission, Infectious"[Mesh]) OR ("Communicable Diseases, Emerging"[Mesh])) OR (transmission[Title/Abstract])) OR (re-introduction[Title/Abstract])) OR (re-establishment[Title/Abstract])) OR (re-emergence[Title/Abstract])) OR (re-surgence[Title/Abstract]))) AND  #3 ((china) OR (chinese mainland) OR (Taiwan) OR (Hongkong) OR (Macao))  #4 (#1 AND #2AND #3) Filters: from 2010 - 2023 |
| Web of science | #1 TS=(Malaria) OR TS=(Malaria, Vivax) OR TS=(Malaria, Falciparum)  #2 TS=(re-introduction) OR TS=(re-establishment) OR TS=(re-emergence) OR TS=(re-surgence)  #3 TS=(china) OR TS=(chinese mainland) OR TS=(Taiwan) OR TS=(Hongkong) OR TS=(Macao)  #4 (2023 or 2022 or 2021 or 2020 or 2019 or 2018 or 2017 or 2016 or 2015 or 2014 or 2013 or 2012 or 2011 or 2010)  #5 (#1 AND #2 AND #3 AND #4) |
| Cochrane Library | #1 Keyword=malaria OR malaria, Vivax OR malaria, Falciparum  #2 Keyword=transmission OR re-introduction OR re-establishment OR re-emergence OR re-surgence  #3 Keyword=China OR Chinese OR Hongkong OR Taiwan OR Macao  #4 (#1 AND #2AND #3) |
| China National Knowledge Infrastructure Database (CNKI) | #1 Topics=malaria OR Malaria, Vivax OR Malaria, Falciparum  #2 Topics=transmission OR re-transmission OR re-introduction OR re-emergence  #3 #1AND#2 |
| VIP Database for Chinese Technical Periodicals | #1 Title/Keyword=malaria OR Malaria, Vivax OR Malaria, Falciparum  #2 Title/Keyword=transmission OR re-transmission OR re-introduction OR re-emergence  #3 #1AND#2 |
| Wanfang database | #1 Topics=malaria OR Malaria, Vivax OR Malaria, Falciparum  #2 Topics=transmission OR re-transmission OR re-introduction OR re-emergence  #3 #1AND#2 |

**
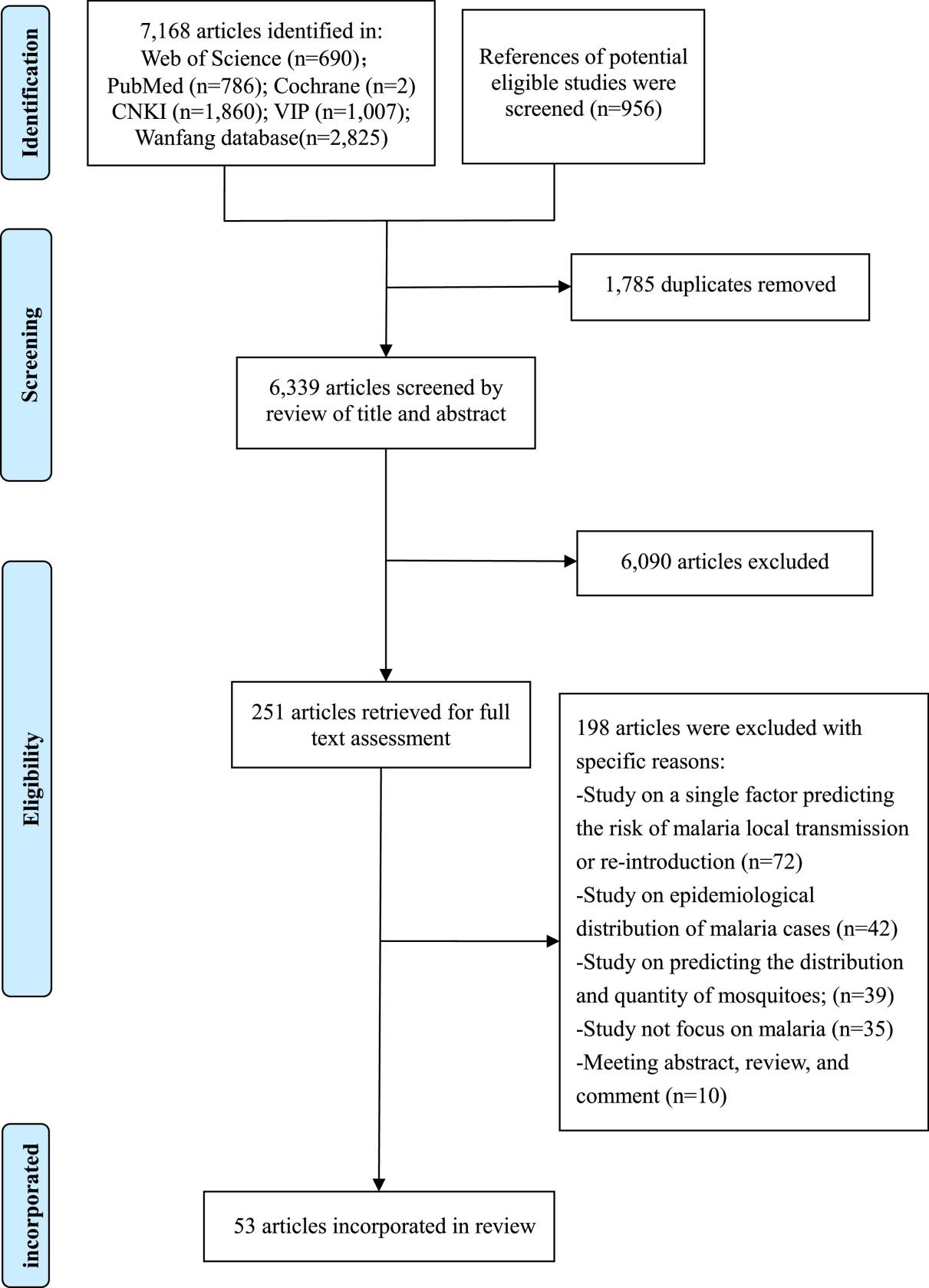
**

**Figure S2.** A summary flow of study selection for research on the risk of malaria local transmission and re-introduction in China from pre-elimination to elimination.

Each record was independently screened by two researchers, first by title and abstract, then by full texts of potentially relevant studies. Any discordance was resolved by discussion or consultation with a third researcher when required.

In the two systematic reviews, indicators to assess the risk of local malaria transmission and reintroduction were extracted and categorized into six domains: environmental and meteorological factors, historical epidemiology factors, vector-related factors, sociodemographic information, surveillance and response-related factors, and population migration factors. A total of 38 indicators used to assess the domain of malaria surveillance and response were extracted and summarized (Online Supplemental Appendix 2 Table S3).

**Table S3.** Malaria surveillance and response factors in post-elimination settings identified through two systematic reviews [1, 2].

| Factors | Number | Indicators |
| --- | --- | --- |
| Mosquito vector surveillance | 2 | Number of vector surveillance sites |
|  |  | Implementation status of vector surveillance and control measures |
| Case detection capacity | 5 | Port diagnostic detection capacity |
|  |  | Incubation period of imported malaria cases |
|  |  | Proportion of patients with unexplained fever that are tested for Plasmodium |
|  |  | Blood testing rate for imported malaria cases |
|  |  | Number of active case surveillance activities |
| Case management capacity | 11 | Proportion of suspected cases tested by the laboratory |
|  |  | Standardized treatment rate of imported malaria cases |
|  |  | Proportion of imported malaria cases reported within 1 day |
|  |  | Proportion of imported malaria case checked and epidemiological investigations completed within 3 days |
|  |  | Proportion of imported malaria cases for which outbreak investigations and case management are completed within 7 days |
|  |  | The timeliness of case management |
|  |  | Proportion of imported malaria cases diagnosed with malaria at the first visit by medical institutions |
|  |  | Correctness of imported malaria “parasite species” identification |
|  |  | Assessment score of diagnostic and treatment skills in medical institutions |
|  |  | Time to achieve diagnostic and treatment skill standards in medical institutions |
|  |  | Interval between the first medical visit and diagnosis |
| Resource stockpiles | 8 | Funding for malaria surveillance and response |
|  |  | Adequate stockpiles of malaria treatment drugs |
|  |  | Number of microscopy stations |
|  |  | Equipment configuration status at grassroots CDCs |
|  |  | Number of microscopes per township |
|  |  | Number of malaria surveillance and response staff per 1,000 population |
|  |  | Number of trainees in malaria surveillance and response training |
|  |  | Status of antimalarial drug stockpiles |
| Government policy support | 7 | Government attention to the risk of malaria re-transmission |
|  |  | Number of work supervision |
|  |  | Number of sentinel hospitals |
|  |  | Whether a malaria surveillance and response leading group has been established |
|  |  | Whether malaria surveillance and response implementation plans have been issued |
|  |  | Establishment of multi-sectoral joint mechanisms |
|  |  | Malaria reporting system coverage rate |
| Publicity and education | 5 | Level of malaria knowledge among the population |
|  |  | Active medical-seeking awareness of inbound personnel |
|  |  | Awareness of diagnosis and treatment among medical staff |
|  |  | Number of malaria awareness-raising materials distributed |
|  |  | Interval between illness onset and the first medical visit |

**References**

1. Lu G, Zhang D, Chen J, Cao Y, Chai L, Liu K, et al. Predicting the risk of malaria re-introduction in countries certified malaria-free: a systematic review. Malaria journal. 2023; 22(1):175.

2. Lu G, Zhao L, Chai L, Cao Y, Chong Z, Liu K, et al. Assessing the risk of malaria local transmission and re-introduction in China from pre-elimination to elimination: A systematic review. Acta tropica. 2024; 249:107082.
