# Supplementary material for: Building a tool to assess malaria surveillance and response capacity in malaria post-elimination contexts: a modified and dual-weighted Delphi approach
Source: Infect Dis Poverty. 2025 Dec 26;14:127. doi: 10.1186/s40249-025-01401-w (PMC12742199; doi:10.1186/s40249-025-01401-w)
Supplement: Supplementary file 3 — Supplementary Material 3. [file 40249_2025_1401_MOESM3_ESM.docx]

**Appendix 3**

**Method and results of the agreement stage and tool development stage**

Process of contacting and selecting the experts

We contacted the potential participants by telephone to explain the purpose of the study and collect background information. Those participants who met the inclusion criteria were sorted according to their primary areas of specialization, and all study team members reviewed each participant’s expression of interest and resume. The study team discussed and considered a range of issues when ranking and selecting panellists, including their years of experience, the representativeness of primary health facilities, relevant experience with malaria surveillance, and the experts’ potential to add to the range of viewpoints in the panel.

Statistical analysis of the two rounds of Delphi

The analyses included calculating the mean, standard deviation, and coefficient of variation for each indicator’s importance and feasibility scores. The recovery rate (RR) was calculated to test the engagement of the experts, and a >70% of RR indicated effective consultation[1]. The degree of expert authority is expressed by the authoritative coefficient (Cr), which is determined by the familiarity with the indicators (Cs) and the judgement basis of the indicators (Ca). The calculation formula for calculating Cr was Cr = (Cs + Ca)/2. A Cr ≥0.70 is generally considered reliable, reflecting a high degree of expert authority in the field[2]. The *CV* reflects the degree of coordination in the evaluation of a specific indicator, with a smaller value indicating less divergence among the experts regarding that indicator. A *CV* of less than 0.25 is generally acceptable [2]. Kendall’s coefficient of concordance *W* statistic is a nonparametric statistical method that is used to calculate the expert coordination coefficient *W* [3]. SPSSAU(25) was employed to calculate the weights of the indicators.

**Table S1.** The boundary values of the two rounds of inclusion and exclusion of indicators.

| Round | Importance | | | Feasibility | | |
| --- | --- | --- | --- | --- | --- | --- |
|  | Arithmetic mean | *CV* | Perfect scores | Arithmetic mean | *CV* | Perfect scores |
| 1 | 4.312 | 0.189 | 51.11 | 4.015 | 0.209 | 31.367 |
| 2 | 4.451 | 0.174 | 60.109 | 4.203 | 0.195 | 39.694 |

**Table S2.** Overview of the Analytic Hierarchy Process (AHP) [4].

| Steps | Description |
| --- | --- |
| 1.Establish Hierarchical Structure | Build a top-down hierarchical model to visually represent the indicator system, consisting of the Objective Layer (assessment of malaria surveillance and response capacity in the post-elimination phase), the Criterion Layer (6 domains), and the Solution Layer (34 indicators; see Figure S1). |
| 2.Construct Judgement Matrix | Use the Saaty scale (1-9) to compare the relative importance of indicators at each level (see Table S3). |
| 3.Calculate Weights and Consistency Check | Compute the weights of indicators at each level and perform a consistency check to ensure logical correctness and reliability of expert judgements. |
| 4.Hierarchical Total Ranking | Calculate the combined weights of the entire indicator system, representing the relative importance of all factors relative to the objective layer. |

**Table S3.** Pairwise comparison scale used in the AHP^a^ for determining the weights of indicators in the malaria surveillance and response capacity assessment framework in post-elimination settings [4].

| Intensity of importance | Definition | Explanation |
| --- | --- | --- |
| 1 | Both elements have equal importance | Both elements contribute equally to the criterion considered |
| 3 | Moderately higher importance of row element  (RE) as compared to column element (CE) | Experience and judgement reveal a slight preference of row element (RE) over column element (CE) |
| 5 | Higher importance of RE as compared to CE | Experience and judgement reveal a strong preference of RE over CE |
| 7 | Much higher importance of RE as compared to CE | RE is very strongly favoured over CE, and its dominance has been demonstrated in practice |
| 9 | Complete dominance in terms of importance  of RE over CE | The evidence favouring RE over CE is of the highest possible order |
| 2, 4, 6, 8 (Intermediate values) | Intermediate position between two assessments | |
| 1/2, 1/3, 1/4,…1/9 (reciprocals) | When CE is compared with RE, it receives the reciprocal value of the RE/CE comparison | |
|  |  | |

^a^ The Saaty scale is used to evaluate the scale values corresponding to the mean differences between two indicators, thereby determining the relative importance of each item within each hierarchical level.

**Table S4.** Overview of the Entropy Method.[5]

| Steps | Description |
| --- | --- |
| 1. Data Standardization | Standardize the raw data to ensure comparability across indicators. Normalization: Transform data into a uniform scale (e.g., 0 to 1) to eliminate unit differences. |
| 2. Calculate Proportions | Convert the standardized data into proportions for each indicator. This step ensures that the sum of proportions for each indicator across all samples equals 1. |
| 3.Calculate Entropy Value | Compute the entropy value for each indicator to assess its information content. Entropy measures how evenly the values are distributed. Higher entropy indicates less variability and thus less information content. |
| 4. Calculate Difference Coefficient | Determine the degree of variation for each indicator by subtracting the entropy from 1. A higher difference coefficient indicates greater variability and more contribution to the evaluation. |
| 5. Calculate Weights | Allocate weights to each indicator based on their difference coefficients. Indicators with higher variability (higher difference coefficients) will receive greater weights in the evaluation. |

**Table S5.** Sources of indicators for the first round of Delphi questionnaires.

| Systematic reviews | Panel meeting |
| --- | --- |
| 1.1 Proportion of cases detected in entry quarantine |  |
| 1.2 Proportion of patients with unexplained fever that are tested for Plasmodium |  |
| 1.3 Proportion of blood samples collected at blood donor centre that are tested for Plasmodium |  |
| 1.4 The interval between entry and confirmation of malaria cases |  |
|  | 1.5 Proportion of suspected cases tested by the laboratory |
|  | 1.6 Proportion of service-delivery points that have been carried out to surveillance drug resistance |
| 1.7 Number of vector surveillance sites |  |
| 1.8 Regularity of vector surveillance activities |  |
|  | 1.9 Proportion of vector surveillance sites that have been carried out to surveillance insecticide resistance |
| 1.10 Proportion of service-delivery points (including CDC and health institutions) that are included in the surveillance system |  |
|  | 2.1 Proportion of reported cases to actual cases in the malaria surveillance system |
|  | 2.2 Proportion of confirmed cases with completed epidemiological investigations and submitted reports |
| 2.3 The timeliness of imported malaria cases reported within 1 day |  |
| 2.4 The timeliness of imported malaria case checked and epidemiological investigations completed within 3 days |  |
| 2.5 The timeliness of imported malaria cases for which outbreak investigations and case management are completed within 7 days |  |
|  | 2.6 Completeness of reporting of core variables in malaria surveillance systems |
|  | 2.7 Consistency of reporting core variables in the National Notifiable Disease Reporting System and the Parasitic Diseases Information Reporting Management System |
|  | 2.8 Malaria epidemiology reporting annually based on surveillance data |
|  | 3.1 Flexibility of the information management system, with straightforward procedures for making revisions (e.g., adding indicators, accommodating changes in case definitions or technology) |
|  | 3.2 Stability and full operation of the information management system with planned and budgeted maintenance |
|  | 3.3 Use of standardized data formats in the information management system, enabling integration with other data sources |
|  | 3.4 The information management system includes functionality for visualizing data in a dashboard |
| 4.1 Number of malaria control staff per 1,000 population |  |
|  | 4.2 Number of vector surveillance staffs per 1,000 population at the township level |
| 4.3 Number of microscopists per 1,000 population |  |
| 4.4 Number of microscopes/rapid diagnostic tests per 1,000 population |  |
| 4.5 Adequate stockpiles of malaria treatment drugs |  |
| 4.6 National funding for malaria surveillance |  |
| 5.1 Proportion of imported malaria cases diagnosed with malaria at the first visit |  |
| 5.2 Correctness of “imported malaria” diagnosis |  |
| 5.3 Correctness of imported malaria “parasite species” identification |  |
| 5.4 Interval between the first medical visit and diagnosis |  |
| 5.5 Standardized treatment rate of imported malaria cases |  |
|  | 6.1 Imported malaria managed as a Category B infectious disease |
| 6.2 Existence of a designated malaria surveillance authority |  |
| 6.3 Establishment of multisectoral joint mechanisms |  |
| 6.4 Frequency of lower-level malaria surveillance units has been supervised |  |
|  | 6.5 Frequency of malaria surveillance data has been reviewed for quality |
| 6.6 Frequency of knowledge and skills training for malaria prevention and control staff |  |
| 6.7 Frequency of knowledge and skills training for medical staff |  |
| 6.8 Number of malaria awareness-raising materials distributed |  |
|  | 6.9 Frequency of public lectures on malaria |
| 6.10 Level of malaria knowledge among the population |  |
| 6.11 Coverage rate of anti-mosquito facilities and tools |  |
| 6.12 Interval between illness onset and the first medical visit |  |

**Table S6.** Domains and indicators used in the two rounds of Delphi consultation and the final constructed assessment framework for malaria surveillance and response capacity in post-elimination settings.

| The indexes for the first round of Delphi | The indexes for the second round of Delphi | The final constructed assessment framework |
| --- | --- | --- |
| **Surveillance system coverage and performance** | **Surveillance system coverage and performance** | **Surveillance system coverage and performance** |
| 1.1 Proportion of cases detected in entry quarantine ^a^ |  |  |
| 1.2 Proportion of patients with unexplained fever that are tested for Plasmodium ^a^ |  |  |
| 1.3 Proportion of blood samples collected at blood donor centre that are tested for Plasmodium ^a^ |  |  |
| 1.4 The interval between entry and confirmation of malaria cases | 1.1 The interval between entry and confirmation of malaria cases ^c^ |  |
| 1.5 Proportion of suspected cases tested by the laboratory | 1.2 Proportion of suspected cases tested by the laboratory | 1.1 Proportion of suspected cases tested by the laboratory |
| 1.6 Proportion of service-delivery points that have been carried out to surveillance drug resistance ^a^ |  |  |
| 1.7 Number of vector surveillance sites ^a^ |  |  |
| 1.8 Regularity of vector surveillance activities | 1.3 Regularity of vector surveillance activities | 1.2 Regularity of vector surveillance activities |
| 1.9 Proportion of vector surveillance sites that have been carried out to surveillance insecticide resistance ^a^ |  |  |
| 1.10 Proportion of service-delivery points (including CDC and health institutions) that are included in the surveillance system ^a^ |  |  |
|  |  | 1.3 Imported malaria reported and managed as a Category B infectious disease (revised from 6.1 in the second round of Delphi) |
|  |  | 1.4 Existence of a designated malaria surveillance authority (revised from 6.2 in the second round of Delphi) |
|  |  | 1.5 Establishment of multisectoral joint mechanisms  (revised from 6.3 in the second round of Delphi) |
| **The quality and use of the surveillance data** | **The quality and use of the surveillance data** | **The quality and use of the surveillance data** |
| 2.1 Proportion of reported cases to actual cases in the malaria surveillance system | 2.1 Proportion of reported cases to actual cases in the malaria surveillance system | 2.1 Proportion of reported cases to actual cases in the malaria surveillance system |
| 2.2 Proportion of confirmed cases with completed epidemiological investigations and submitted reports | 2.2 Proportion of confirmed cases with completed epidemiological investigations and submitted reports | 2.2 Proportion of confirmed cases with completed epidemiological investigations and submitted reports |
| 2.3 The timeliness of imported malaria cases reported within 1 day ^b^ | 2.3 Proportion of imported malaria cases reported within 1 day | 2.3 Proportion of imported malaria cases reported within 1 day |
| 2.4 The timeliness of imported malaria case checked and epidemiological investigations completed within 3 days ^b^ | 2.4 Proportion of imported malaria case checked and epidemiological investigations completed within 3 days | 2.4 Proportion of imported malaria case checked and epidemiological investigations completed within 3 days |
| 2.5 The timeliness of imported malaria cases for which outbreak investigations and case management are completed within 7 days ^b^ | 2.5 Proportion of imported malaria cases for which outbreak investigations and case management are completed within 7 days | 2.5 Proportion of imported malaria cases for which outbreak investigations and case management are completed within 7 days |
| 2.6 Completeness of reporting of core variables in malaria surveillance systems | 2.6 Completeness of reporting of core variables in malaria surveillance systems | 2.6 Completeness of reporting of core variables in malaria surveillance systems |
| 2.7 Consistency of reporting core variables in the National Notifiable Disease Reporting System and the Parasitic Diseases Information Reporting Management System | 2.7 Consistency of reporting core variables in the National Notifiable Disease Reporting System and the Parasitic Diseases Information Reporting Management System | 2.7 Consistency of reporting core variables in the National Notifiable Disease Reporting System and the Parasitic Diseases Information Reporting Management System |
| 2.8 Malaria epidemiology reporting annually based on surveillance data ^b^ | 2.8 Malaria epidemiology reporting monthly based on surveillance data ^d^ | 2.8 Regular malaria epidemiology reporting based on surveillance data |
| **The functioning of the surveillance information management system** | **The functioning of the surveillance information management system** | **The functioning of the surveillance information management system** |
| 3.1 Flexibility of the information management system, with straightforward procedures for making revisions (e.g., adding indicators, accommodating changes in case definitions or technology) | 3.1 Flexibility of the information management system, with straightforward procedures for making revisions (e.g., adding indicators, accommodating changes in case definitions or technology) | 3.1 Flexibility of the information management system, with straightforward procedures for making revisions (e.g., adding indicators, accommodating changes in case definitions or technology) |
| 3.2 Stability and full operation of the information management system with planned and budgeted maintenance | 3.2 Stability and full operation of the information management system with planned and budgeted maintenance | 3.2 Stability and full operation of the information management system with planned and budgeted maintenance |
| 3.3 Use of standardized data formats in the information management system, enabling integration with other data sources | 3.3 Use of standardized data formats in the information management system, enabling integration with other data sources | 3.3 Use of standardized data formats in the information management system, enabling integration with other data sources |
| 3.4 The information management system includes functionality for visualizing data in a dashboard | 3.4 The information management system includes functionality for visualizing data in a dashboard | 3.4 The information management system includes functionality for visualizing data in a dashboard |
| **The availability and adequacy of resources** | **The availability and adequacy of resources** | **The availability and adequacy of resources** |
| 4.1 Number of malaria control staff per 1,000 population | 4.1 Number of malaria control staff per 1,000 population | 4.1 Number of malaria control staff per 1,000 population |
| 4.2 Number of vector surveillance staffs per 1,000 population at the township level ^a^ |  |  |
| 4.3 Number of microscopists per 1,000 population | 4.2 Number of microscopists per 1,000 population | 4.2 Number of microscopists per 1,000 population |
| 4.4 Number of microscopes/rapid diagnostic tests per 1,000 population | 4.3 Number of microscopes/rapid diagnostic tests per 1,000 population (divided into 4.3 and 4.4 in the final framework) | 4.3 Number of microscopes per 1,000 population |
|  |  | 4.4 Number of rapid diagnostic tests per 1,000 population ^e^(added from 4.3 in the second round of Delphi) |
| 4.5 Adequate stockpiles of malaria treatment drugs | 4.4 Adequate stockpiles of malaria treatment drugs | 4.5 Adequate stockpiles of malaria treatment drugs |
| 4.6 National funding for malaria surveillance | 4.5 National funding for malaria surveillance ^d^ | 4.6 Actual cost of malaria surveillance and response implementation as a percentage of allocation |
| **Early diagnosis and treatment** | **Early diagnosis and treatment** | **Early diagnosis and treatment** |
| 5.1 Proportion of imported malaria cases diagnosed with malaria at the first visit | 5.1 Proportion of imported malaria cases diagnosed with malaria at the first visit | 5.1 Proportion of imported malaria cases diagnosed with malaria at the first visit |
| 5.2 Correctness of “imported malaria” diagnosis | 5.2 Correctness of “imported malaria” diagnosis | 5.2 Correctness of “imported malaria” diagnosis |
| 5.3 Correctness of imported malaria “parasite species” identification | 5.3 Correctness of imported malaria “parasite species” identification | 5.3 Correctness of imported malaria “parasite species” identification |
|  |  | 5.4 Interval between illness onset and the first medical visit (revised from 6.11 in the second round of Delphi) |
| 5.4 Interval between the first medical visit and diagnosis | 5.4 Interval between the first medical visit and diagnosis | 5.5 Interval between the first medical visit and diagnosis |
| 5.5 Standardized treatment rate of imported malaria cases | 5.5 Standardized treatment rate of imported malaria cases | 5.6 Standardized treatment rate of imported malaria cases |
| **Quality control supervision and training** | **Quality control supervision and training** | **Quality control supervision and training** |
| 6.1 Imported malaria managed as a Category B infectious disease ^b f^ | 6.1 Imported malaria reported and managed as a Category B infectious disease ^d^ (revised to 1.3 in the final framework) |  |
| 6.2 Existence of a designated malaria surveillance authority | 6.2 Existence of a designated malaria surveillance authority ^d^ (revised to 1.4 in the final framework) |  |
| 6.3 Establishment of multisectoral joint mechanisms | 6.3 Establishment of multisectoral joint mechanisms ^d^ (revised to 1.5 in the final framework) |  |
| 6.4 Frequency of lower-level malaria surveillance units supervised ^b^ | 6.4 Frequency of supervision visits by higher administration per year | 6.1 Frequency of supervision visits by higher administration per year |
| 6.5 Frequency of malaria surveillance data has been reviewed for quality ^b^ | 6.5 Frequency of quality reviews of malaria surveillance data (implementation of the “1-3-7” approach ^g^) | 6.2 Frequency of quality reviews of malaria surveillance data (implementation of the “1-3-7” approach) |
| 6.6 Frequency of knowledge and skills training for malaria prevention and control staff | 6.6 Frequency of knowledge and skills training for malaria prevention and control staff | 6.3 Frequency of knowledge and skills training for malaria prevention and control staff |
| 6.7 Frequency of knowledge and skills training for medical staff | 6.7 Frequency of knowledge and skills training for medical staff | 6.4 Frequency of knowledge and skills training for medical staff |
| 6.8 Number of malaria awareness-raising materials distributed | 6.8 Number of malaria awareness-raising materials distributed ^c^ |  |
| 6.9 Frequency of public lectures on malaria | 6.9 Frequency of public lectures on malaria ^c^ |  |
| 6.10 Level of malaria knowledge among the population | 6.10 Level of malaria knowledge among the population ^d^ | 6.5 Awareness and knowledge of malaria prevention among migrant labours to malaria endemic countries |
| 6.11 Coverage rate of anti-mosquito facilities and tools ^a^ |  |  |
| 6.12 Interval between illness onset and the first medical visit | 6.11 Interval between illness onset and the first medical visit ^d^ (revised to 5.4 in the final framework) |  |

^a^ Deleted after the first round of Delphi

^b^ Revised after the first round of Delphi

^c^ Deleted after the second round of Delphi

^d^ Revised after the second round of Delphi

^e^ Added after the second round of Delphi

^f^ Category B infectious disease: Diseases that pose significant threats to human health and safety, potentially causing substantial economic losses and social impacts [6]. For Category B infectious diseases, online reporting within 24 hours of detection is mandatory, with management measures including patient treatment, transmission control, and close contact tracking [7].

^g^ "1-3-7" approach: A malaria surveillance and response strategy developed in China. It mandates that malaria cases be reported within 1 day and confirmed and investigated within 3 days and that public health responses to prevent further transmission occur within 7 days [8].

**Table S7.** Weights of domains and indicators with the combination of the AHP and entropy methods.

| **The domain** | **Weights** | **The indicator** | **Weights** |
| --- | --- | --- | --- |
| **Surveillance system coverage and performance** | 0.2400 | Proportion of suspected cases tested by the laboratory | 0.0751 |
|  |  | Regularity of vector surveillance activities | 0.0091 |
|  |  | Imported malaria reported and managed as a Category B infectious disease | 0.1101 |
|  |  | Existence of a designated malaria surveillance authority | 0.0294 |
|  |  | Establishment of multisectoral joint mechanisms | 0.0163 |
| The quality and use of the surveillance data | 0.3710 | Proportion of reported cases to actual cases in the malaria surveillance system | 0.0218 |
|  |  | Proportion of confirmed cases with completed epidemiological investigations and submitted reports | 0.1153 |
|  |  | Proportion of imported malaria cases reported within 1 day | 0.0501 |
|  |  | Proportion of imported malaria case checked and epidemiological investigations completed within 3 days | 0.0777 |
|  |  | Proportion of imported malaria cases for which outbreak investigations and case management are completed within 7 days | 0.0325 |
|  |  | Completeness of reporting of core variables in malaria surveillance systems | 0.0501 |
|  |  | Consistency of reporting core variables in the National Notifiable Disease Reporting System and the Parasitic Diseases Information Reporting Management System | 0.0151 |
|  |  | Regular malaria epidemiology reporting based on surveillance data | 0.0083 |
| The functioning of the surveillance information management system | 0.0973 | Flexibility of the information management system, with straightforward procedures for making revisions (e.g., adding indicators, accommodating changes in case definitions or technology) | 0.0076 |
|  |  | Stability and full operation of the information management system with planned and budgeted maintenance | 0.0476 |
|  |  | Use of standardized data formats in the information management system, enabling integration with other data sources | 0.0297 |
|  |  | The information management system includes functionality for visualizing data in a dashboard | 0.0123 |
| **The availability and adequacy of resources** | 0.0375 | Number of malaria control staff per 1000 population | 0.0026 |
|  |  | Number of microscopists per 1000 population | 0.0009 |
|  |  | Number of microscopes per 1000 population | 0.0026 |
|  |  | Number of rapid diagnostic tests per 1000 population | 0.0070 |
|  |  | Adequate stockpiles of malaria treatment drugs | 0.0174 |
|  |  | Actual cost of malaria surveillance and response implementation as a percentage of allocation | 0.0070 |
| **Early diagnosis and treatment** | 0.1571 | Proportion of imported malaria cases diagnosed with malaria at the first visit | 0.0087 |
|  |  | Correctness of “imported malaria” diagnosis | 0.0145 |
|  |  | Correctness of imported malaria “parasite species” identification | 0.0087 |
|  |  | Interval between illness onset and the first medical visit | 0.0058 |
|  |  | Interval between the first medical visit and diagnosis | 0.1131 |
|  |  | Standardized treatment rate of imported malaria cases | 0.0063 |
| **Quality control supervision and training** | 0.0973 | Frequency of supervision visits by higher administration per year | 0.0052 |
|  |  | Frequency of quality reviews of malaria surveillance data (implementation of the “1-3-7” approach) | 0.0128 |
|  |  | Frequency of knowledge and skills training for malaria prevention and control staff | 0.0637 |
|  |  | Frequency of knowledge and skills training for medical staff | 0.0148 |
|  |  | Awareness and knowledge of malaria prevention among migrant labours to malaria endemic countries | 0.0008 |


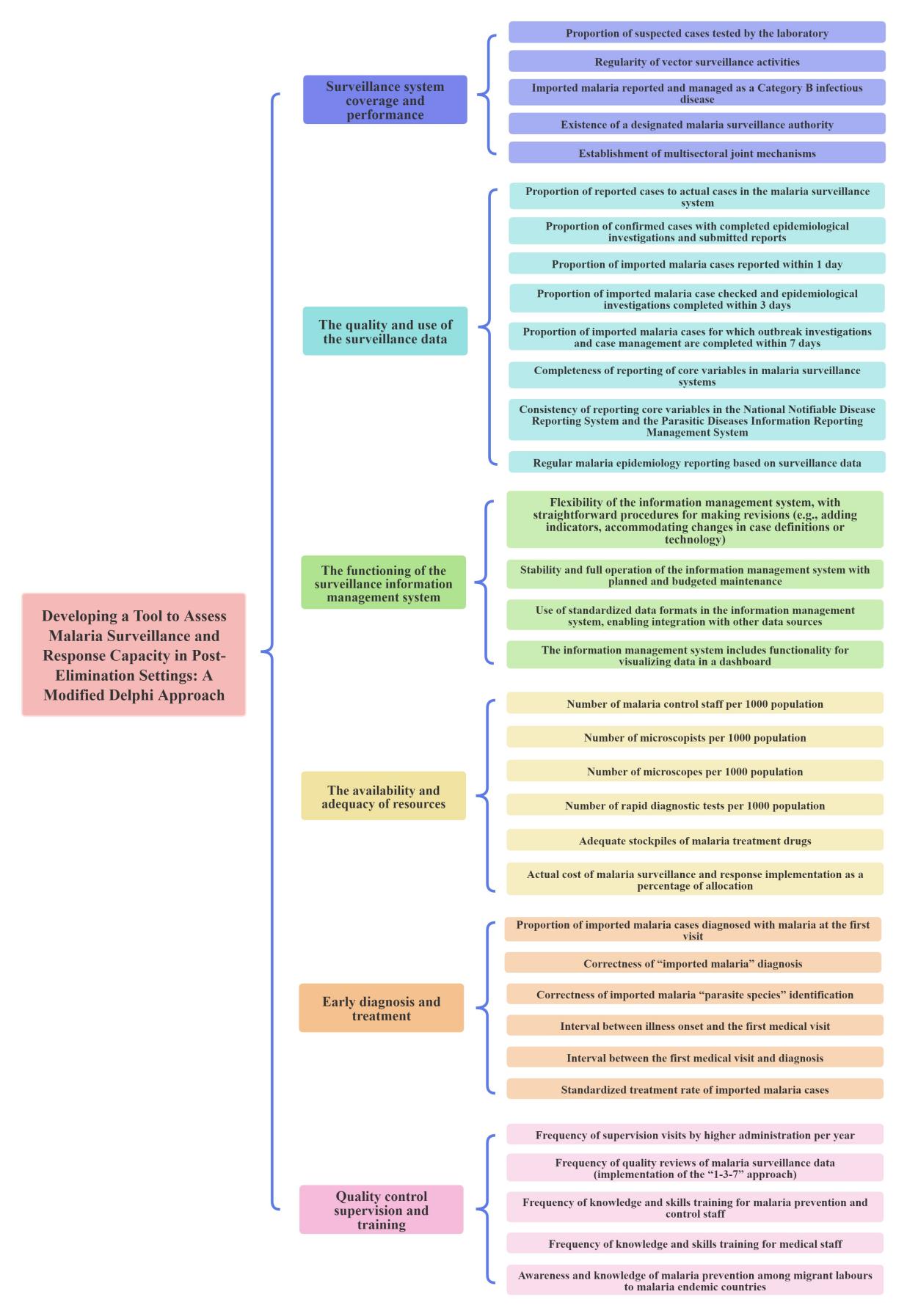


**Figure S1.** Framework of the six domains and 34 indicators for assessing malaria surveillance and response capacity in post-elimination settings.

**References**

1. Wu JL, Pang SQ, Jiang XM, et al. Which mothers' breastfeeding behaviours within six weeks postpartum do health workers need to notice? Developing an indicator system based on the Delphi method and analytic hierarchy process. Midwifery. 2023;119:103603.

2. Zhao ZG, Cheng JQ, Xu SL, Hou WL, Richardus JH. A quality assessment index framework for public health services: a Delphi study. Public Health. 2015;129(1):43-51.

3. Lei L, Richards JS, Li ZH, Gong YF, Zhang SZ, Xiao N. A framework for assessing local transmission risk of imported malaria cases. Infect Dis Poverty. 2019;8(1):43.

4. Saaty T, Vargas L. Models, Methods, Concepts & Applications of the Analytic Hierarchy Process 2012. https://doi.org/10.1007/978-1-4614-3597-6_2. Accessed 2 July 2024.

5. Zhu Y, Tian D, Yan F. Effectiveness of Entropy Weight Method in Decision-Making. 2020;2020(1):3564835.

6. Chinese Center for Disease Control and Prevention (China CDC). Categories of infectious diseases 2022. Available from: <https://en.chinacdc.cn/health_topics/infectious_diseases/202203/t20220301_257279.html>. Accessed 20 July 2025.

7. National Health Commission of the People's Republic of China. Measures for the Administration of Surveillance and Reporting of Information on Public Health Emergencies and Infectious Disease Outbreaks 2022. <http://www.nhc.gov.cn/fzs/s3576/201808/6d00c158844f42c5bcf94993bffa665a.shtml>. Accessed 20 July 2025.

8. Lu G, Liu Y, Beiersmann C, Feng Y, Cao J, Müller O. Challenges in and lessons learned during the implementation of the 1-3-7 malaria surveillance and response strategy in China: a qualitative study. Infect Dis Poverty. 2016;5(1):94.
